# Supplementary material for: Fabrication, Structure and Functional Characterizations of pH-Responsive Hydrogels Derived from Phytoglycogen
Source: Foods. 2021 Nov 1;10(11):2653. doi: 10.3390/foods10112653 (PMC8621403; doi:10.3390/foods10112653)
Supplement: Supplementary file 1 [file foods-10-02653-s001.zip › foods-1412630-supplementary.pdf]

Table S1 Gelation process of different hydrogels.

	Time (h)					
	1	2	3	4	5	6
Gel 1	+	+	+	+	+	+
Gel 2	-	+	+	+	+	+
Gel 3	-	-	-	+	+	+
Gel 4	-	-	-	-	+	+
Gel 5	-	-	-	-	-	+
